# Fidelity Variants of RNA Dependent RNA Polymerases Uncover an Indirect, Mutagenic Activity of Amiloride Compounds

**DOI:** 10.1371/journal.ppat.1001163

**Published:** 2010-10-28

**Authors:** Laura I. Levi, Nina F. Gnädig, Stéphanie Beaucourt, Malia J. McPherson, Bruno Baron, Jamie J. Arnold, Marco Vignuzzi

**Affiliations:** 1 Institut Pasteur, Viral Populations and Pathogenesis Lab and CNRS URA3015, Paris, France; 2 Institut Pasteur, Plate Forme de Biophysique des Macromolécules et de leurs Interactions, Paris, France; 3 Department of Biochemistry and Molecular Biology, Pennsylvania State University, University Park, Pennsylvania, United States of America; Washington University School of Medicine, United States of America

## Abstract

In a screen for RNA mutagen resistance, we isolated a high fidelity RNA dependent RNA polymerase (RdRp) variant of Coxsackie virus B3 (CVB3). Curiously, this variant A372V is also resistant to amiloride. We hypothesize that amiloride has a previously undescribed mutagenic activity. Indeed, amiloride compounds increase the mutation frequencies of CVB3 and poliovirus and high fidelity variants of both viruses are more resistant to this effect. We hypothesize that this mutagenic activity is mediated through alterations in intracellular ions such as Mg^2+^ and Mn^2+^, which in turn increase virus mutation frequency by affecting RdRp fidelity. Furthermore, we show that another amiloride-resistant RdRp variant, S299T, is completely resistant to this mutagenic activity and unaffected by changes in ion concentrations. We show that RdRp variants resist the mutagenic activity of amiloride via two different mechanisms: 1) increased fidelity that generates virus populations presenting lower basal mutation frequencies or 2) resisting changes in divalent cation concentrations that affect polymerase fidelity. Our results uncover a new antiviral approach based on mutagenesis.

## Author Summary

RNA viruses have extreme mutation frequencies, due in large part to the erroneous nature of the viral RNA dependent RNA polymerases (RdRp) that replicate their genomes. Since RdRp lack proofreading and repair mechanisms, the use of base analogs as RNA mutagens to increase lethal mutations and extinguish the virus population is a promising antiviral strategy. Recently, a screen for resistance to this antiviral treatment identified a higher fidelity RdRp variant of poliovirus, indicating that RdRp fidelity can be modulated by single amino acid substitutions. To extend these observations to other viruses, we performed a similar screen using Coxsackie virus B3 (CVB3). We identified a new high fidelity RdRp variant which was also resistant to amiloride compounds that have no known mutagenic activity. Using wild type and RdRp fidelity variants of poliovirus and CVB3, we show that amiloride compounds do have mutagenic activity and act on RNA virus populations indirectly, by altering intracellular ion concentrations that affect polymerase fidelity. Our results identify a new means of targeting viruses through increases in mutation frequency using non-nucleoside compounds that alter the cellular environment in which the virus replicates.

## Introduction

Amiloride and its derivatives are potassium-sparing diuretics used to treat hypertension and to prevent hypokalemia associated with congestive heart failure. These compounds act by inhibiting epithelial Na^+^ channels and the Na^+^/H^+^, Na^+^/Ca^2+^ and Na^+^/Mg^2+^ antiport functions [1], [2]. Due to its relatively low toxicity, the antiviral properties of amiloride is being explored. This compound inhibits the viroporins of coronaviruses, flaviviruses and retroviruses [3], [4], [5], [6]. More recently, amiloride was shown to exert an antiviral effect on rhinovirus (common cold) and Coxsackie virus B3 (CVB3, viral myocarditis) by directly affecting virus replication or release [7], [8]. In this study, Harrison *et al*. isolated two viral RNA dependent RNA polymerase (RdRp) mutants of CVB3 that were more resistant to amiloride than wild type virus. The mechanism for this resistance remains unclear.

We isolated one of these same CVB3 RdRp mutants in a screen for resistance to RNA mutagens with the goal of identifying higher fidelity RdRp variants. RNA mutagens such as ribavirin, 5-fluorouracil and 5-azacytidine are base analogs that are incorrectly inserted into the genome by the RdRp during replication and result in the accumulation of lethal mutations over several passages, a process referred to as lethal mutagenesis[9], [10], [11], [12], [13]. A similar screen previously identified a higher fidelity variant of poliovirus, suggesting that the intrinsic fidelity of RdRps can be modulated, despite their lack of proofreading functions [14], [15]. Poliovirus RdRp fidelity variants have since proven to be useful tools for studying the role of genetic diversity in virus fitness and virulence, and have shown promise in improving vaccine attenuation and genetic stability [16], [17], [18]. In order to extend these observations to other medically relevant viruses, we performed a screen for high fidelity variants of CVB3 by selecting for resistance to the mutagenic base analogs, ribavirin and 5-azacytidine. Here we identify a CVB3 variant with higher fidelity that maps to a different region of the RdRp than the previously described position 64 of poliovirus [18]. Since the same mutation confers resistance to RNA mutagens and to amiloride, we hypothesized that amiloride has a previously unknown mutagenic activity. In this report we provide the first evidence for an indirect RNA mutagenic activity for the amiloride compounds and show that the amiloride resistant CVB3 RdRp variants resist this mutagenic activity through two different mechanisms.

## Results

### Isolation of RNA mutagen resistant Coxsackie virus (CVB3) with a high fidelity RdRp

**Figure 1:**
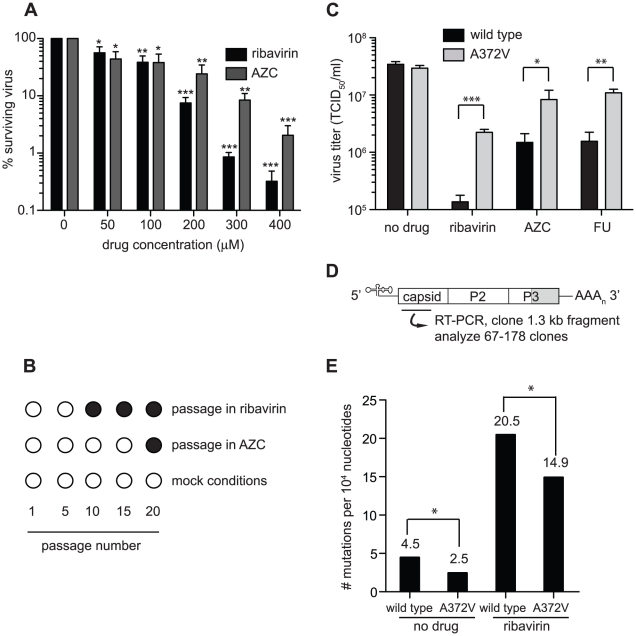
Isolation of an RNA mutagen resistant CVB3 with a high fidelity RdRp. (**A**) Optimization of the RNA mutagen treatment concentrations to select for resistant CVB3 variants. HeLa cells, treated with different concentrations of either ribavirin or AZC were infected with wild type CVB3 at MOI of 0.01. 48 hours after infection, progeny virus was quantified by TCID_50_ assay. The percentage of viruses surviving treatment (y-axis) with 50, 100, 200, 300 or 400 µM of ribavirin or AZC (x-axis) was determined by dividing the virus titers of treated populations by virus titers in untreated controls (0 µM). Mean values ± S.E.M are shown, N = 4, * P<0.01, ** P<0.001, ***P<0.0001. (**B**) Isolation of RNA mutagen resistant CVB3. CVB3 was passaged 20 times in 50 µM ribavirin, 50 µM AZC or mock treated HeLa cells. Every 5 passages the virus population was sequenced. The emergence of a single point mutation resulting in a A372V change in the RdRp is indicated by a solid circle. (**C**) A372V is resistant to three different base analog RNA mutagens. HeLa cells treated with 300 µM of ribavirin or AZC, or 150 µM FU, were infected with either wild type CVB3 (black bars) or A372V variant (gray bars) at an MOI of 0.01. Control infections (no drug) were also performed. At 48 hours after infection, progeny virus was titered by TCID_50_ assay. Mean virus titers ± S.E.M are shown, N = 8, * P<0.05, ** P<0.01, *** P<0.001. (**D**) Schematic of the viral RNA genome showing the 5′ untranslated region, structural capsid coding region, P2 and P3 region of non structural proteins, including the RdRp (shaded gray) and the 3′ untranslated region. A 1.3 kb region of the viral capsid was RT-PCR amplified and subcloned for sequencing of individual clones to obtain the observed mutation frequencies presented throughout this work. The total number of clones and nucleotides sequenced in each population is shown in Table S1. (**E**) Average mutation frequencies of each viral population shown as the mean number of mutations per 10^4^ nucleotides sequenced. Statistical analysis is described in Methods. * P<0.05 statistical significance of difference between WT and A372V mutation frequencies.

**Figure 2:**
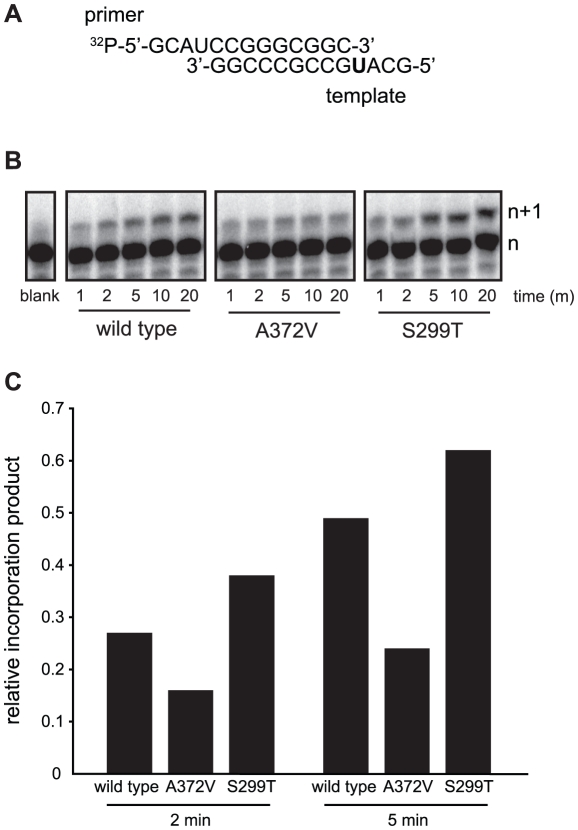
*In vitro* biochemical assays confirm the higher (A372V) and lower (S299T) incorporation fidelities of CVB3 variants. (**A**) A radiolabeled primer/template is used to measure the incorporation of bases in vitro using purified RdRp enzymes, conditions detailed in Methods. The templating U (in bold) permits the quantification of incorporation of the correct (ATP) or incorrect (GTP) nucleotide over time. (**B**) Visualization of GTP mis-incorporation by wild type, A372V and S299T RdRp. The incorporation of GTP is monitored over time (m) as the accumulation of elongation product (n+1) minus blank control. (**C**) Quantification of relative incorporation product at 2 and 5 minutes reaction time.

To determine the conditions in which to generate resistance to RNA mutagens, we treated CVB3 with various concentrations of ribavirin and 5-azacytidine (AZC). Mutagen concentrations above 100 µM decreased viral viability by over 90% (Figure 1A). In order to exert a strong enough selective pressure, without extinguishing the virus population during passage, we serially passaged a large virus population size (10^6^ TCID_50_) in 50 µM of either mutagen. Every five passages, the viral RNA-dependent RNA polymerase (RdRp) region was sequenced (Figure 1B). Wild type CVB3 acquired a new CtoU mutation resulting in an Alanine to Valine change at position 372 of the RdRp (A372V) after passage in both ribavirin (by passage 10) and AZC (by passage 20), that did not arise in untreated control passages. The earlier emergence of this mutation under ribavirin treatment correlated with ribavirin's bias towards GtoA and CtoU transition mutations [19], compared to AZC's bias for CtoG and GtoC transversions [20]. The role of A372V in resistance was confirmed by introducing the mutation back into the CVB3 wild type cDNA infectious clone and comparing the growth of A372V to wild type virus treated with 3 RNA mutagens with different nucleotide structures: ribavirin (300 µM), AZC (300 µM) and 5-fluorouracil (FU, 150 µM). Indeed, A372V was more resistant than wild type virus in each case (Figure 1C). A similar profile of broad resistance to different base analogs was previously observed for the poliovirus G64S high fidelity variant [17], suggesting that A372V is also a higher fidelity RdRp. To confirm this with genetic data, wild type and A372V virus stocks were prepared from infectious clones and used to infect cells that were treated with 400 µM ribavirin, or mock treated. At total cytopathic effect (48 hours after infection), the mutation frequencies and distributions within each population was determined by sequencing a 1.3 kb fragment of the capsid region from individual genomes (Figure 1D). The untreated wild type virus population presented a mutation frequency of 4.5 mutations per 10^4^ nucleotides (Figure 1E and Table S1). The A372V population, on the other hand, presented a mutation frequency that was approximately 2-fold lower than wild type (*P* = 0.0225), thereby confirming the higher fidelity of this variant. As expected, treatment with 400 µM ribavirin significantly increased the mutation frequency of both virus populations (*P*<0.0001); however, the mutation frequency of A372V remained lower than wild type (*P* = 0.039). Furthermore, individual clones in the wild type virus population more often presented multiple mutations compared to A372V (Table S1). These results further confirmed the increased fidelity of the A372V RdRp. To provide further evidence for the increased fidelity of A372V RdRp, an *in vitro* biochemical assay was used to examine the relative levels of misincorporation for both wild type and A372V enzymes. Briefly, *in vitro* reactions were performed using equal amounts of each purified RdRp, saturating concentrations of nucleotide and a radiolabeled RNA primer-template substrate that permits nucleotide addition to be monitored by the extension of end-labeled primer (Figure 2A) [21]. By adding GTP to the reaction, the incorrect incorporation of this nucleotide can be monitored over time by the accumulation of n+1 product. As observed in Figure 2B–C, A372V RdRp consistently incorporated less GMP over time than wild type RdRp. Interestingly, the observed rate of correct nucleotide incorporation for wild type and A372V showed no significant difference, whereas the observed rate of GMP misincorporation showed a 2-fold difference between wild type and A372V RdRp (Table 1), confirming the increased fidelity of the A372V enzyme.

**Table 1:** Incorporation kinetics and relative fidelity of CVB3 RdRp variants.

Enzyme	k_obs_ (s^−1^) correct nt^a^	k_obs_ (s^−1^) incorrect nt^b^	Relative Fidelity^c^
wild type	8.2±0.5	0.006±0.001	1367
A372V	7.5±0.5	0.003±0.001	2500
S299T	7.4±0.2	0.008±0.001	925

a 1 mM ATP, determined from stopped-flow fluorescence assay for AMP incorporation.

b 5 mM GTP, determined from ^32^P-labeled primer-extension assay for GMP misincorporation.

c k_obs correct_/k_obs incorrect_.

### The dual resistance of A372V to RNA mutagens and to amiloride is not a product of increased replicative capacity

**Figure 3:**
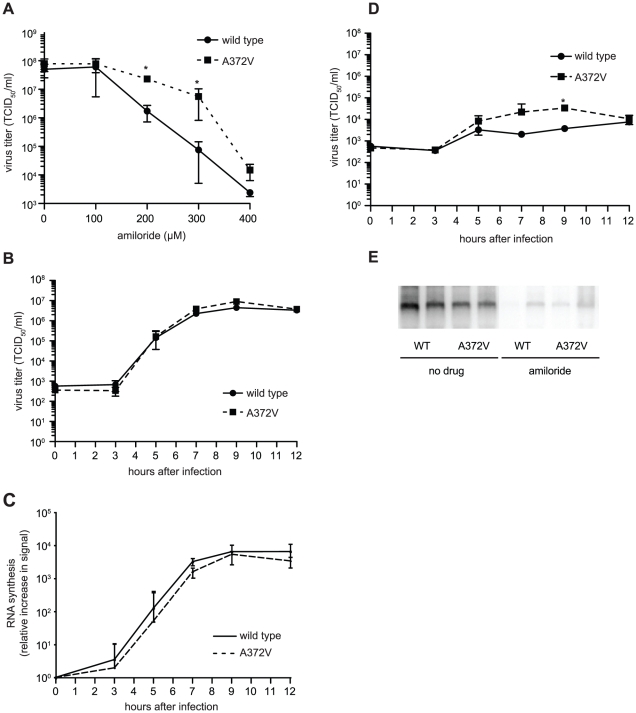
A372V resistance to amiloride does not involve improved replication. (**A**) A372V is more resistant than wild type in increasing concentrations of amiloride. HeLa cells were treated with different concentrations of amiloride and infected with wild type or A372V virus at an MOI of 0.01. At 48 h, viable progeny virus was quantified by TCID_50_ assay. The mean virus titers (TCID_50_/ml) and S.E.M are shown, N = 3, * P<0.05. (**B**) One-step growth kinetics of wild type and A372V viruses. HeLa cells were infected at MOI of 10 and the progeny virus was quantified at different hours after infection by TCID_50_ assay. Mean titers (TCID_50_/ml) ± S.E.M are shown, N = 3, no significant difference found. (**C**) The relative increase in signal from time 0 to indicated time points is shown, values are means of 2 separate northen blots of samples from (B), error bars show range of values (**D**) One-step growth kinetics of wild type and A372V viruses in the presence of 400 µM amiloride. HeLa cells were infected at MOI of 10 and the progeny virus was quantified at different hours after infection by TCID_50_ assay. Mean titers (TCID_50_/ml) ± S.E.M are shown, N = 3, no significant differences found except *, p = 0.03. (**E**) Northern blot analysis of RNA synthesis determined 48 hours after infection with wild type or A372V virus at MOI of 0.01 in the presence or the absence of 400 µM amiloride. Two independent treatment samples per virus are shown.

Curiously, A372V was previously isolated in a screen for resistance to amiloride, a compound with no known mutagenic activity, that was shown to reduce CVB3 titers by partially inhibiting RNA synthesis [8]. Accordingly, A372V consistently replicated to higher titers than wild type at all concentrations of amiloride tested (Figure 3A). A possible explanation for the dual resistance to both RNA mutagens and amiloride is that A372V produces more RNA genomes in the presence of these compounds and would thus titer higher than wild type. To determine whether higher A372V titers in the presence of amiloride were due to increased replicative capacity, one-step growth analysis was performed, in the presence or absence of amiloride. No significant differences in virus production kinetics were observed for wild type and A372V grown in the absence of amiloride (Figure 3B), indicating that increasing fidelity of the CVB3 RdRp did not significantly impact virus multiplication. Furthermore, RNA synthesis of wild type and A372V had similar kinetics and were within the same order of magnitude, as determined by northern blot analysis (Figure 3C). In fact, A372V virus produced slightly less RNA than wild type, yet consistently titered slightly higher because the RNA genomes made contain fewer deleterious mutations. Importantly, differences observed for wild type and A372V one-step growth kinetics in the presence of 400 µM amiloride were not statistically significant, with the exception of one time point (9 hours, p = 0.03) (Figure 3D) and northern blot analysis revealed that both viruses were similarly inhibited in RNA synthesis (Figure 3E). Heightened replicative capacity was thus not responsible for the resistance of A372V to amiloride.

### Amiloride compounds have a RNA mutagenic activity

**Figure 4:**
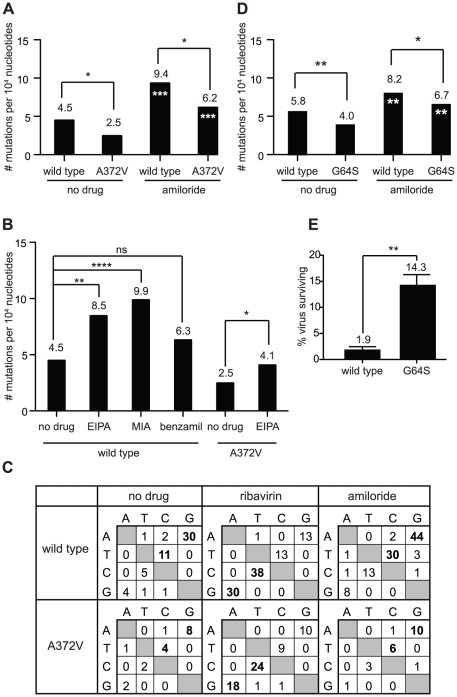
Amiloride has RNA mutagenic activity to which high fidelity RdRp variants of picornaviruses resist. (**A**) The mutation frequencies of wild type and A372V viruses grown in the presence of 400 µM amiloride were determined, shown as the mean number of mutations per 10^4^ nucleotides sequenced. * P<0.05 statistically significant difference between WT and A372V. Asterisks in white indicate statistically significant difference between amiloride-treated virus and the untreated parental population, *** P<0.001. (**B**) Mutations frequencies of wild type and A372V viruses in the presence of other amiloride compounds. HeLa cells were treated with 40 µM of EIPA, 25 µM of MIA or 20 µM of benzamil and were infected with wild type CVB3 virus or A372V variant (EIPA only). Statistical significance of differences in mutation frequencies are indicated. ns = not significant, * P<0.05, ** P<0.005, **** P<0.0001. (**C**) Mutation profiles of wild type and A372V populations grown in the absence of drug or presence of 400 µm ribavirin or amiloride in (A). The most commonly occurring mutations are indicated in bold. (**D**) Mutation frequency of wild type and G64S polioviruses. Viral RNA genomes were extracted following infection of HeLa cells, grown in standard conditions or treated with 400 µM amiloride. A 1.0 kb region of the viral capsid was RT-PCR amplified and subcloned for sequencing of individual clones to obtain the observed mutation frequencies (Table S1). Asterisks in white indicate statistical significance between drug treated populations and the same untreated parental virus. * P<0.05, ** P<0.005. (**E**) The percentage of polioviruses surviving amiloride treatment relative to untreated control populations was determined by plaque assay. The mean values ± S.E.M are shown, N = 3. ** P<0.005.

Since it is unlikely that the same mutation confers resistance to two unrelated antiviral mechanisms, we explored whether amiloride has a previously unknown mutagenic activity. This possibility was not evident, since amiloride is not a base analog such as ribavirin, AZC and FU, whose mutagenic effects result from their direct misincorporation into genomes by the error-prone RdRp. We hypothesized that in addition to inhibiting RNA replication, amiloride compounds increase the virus mutation frequencies of the RNA genomes that are replicated. To address this, wild type and A372V virus infections were performed in the presence of either ribavirin or amiloride, or in the absence of either compound and the mutation frequencies of the resulting populations were determined (Figure 4A). At 400 µM amiloride, the mutation frequency of wild type CVB3 increased from 4.5 to 9.4 mutations per 10^4^ nucleotides (*P* = 0.0005). Similarly, A372V virus also increased mutation frequency, from 2.5 to 6.2 mutations per 10^4^ nucleotides (*P* = 0.0008). However, since the basal mutation frequency of A372V was lower, this increase was better tolerated, explaining the high virus titers observed (Figure 3A). Next, we determined whether the observed mutagenic activity was common to a wider range of amiloride compounds. Wild type virus was treated with the amiloride derivatives EIPA, MIA and benzamil and the mutation frequencies were determined as described above (Figure 4B). Again, the mutation frequencies for wild type CVB3 increased from the basal 4.5 mutations per 10^4^ nucleotides to 8.5 for EIPA (*P* = 0.0028) and to 9.9 for MIA (*P*<0.0001). Although a tendency towards increase was observed for benzamil (6.3 mutations per 10^4^ nucleotides), no statistically significant difference was established (*P* = 0.105). Furthermore, treatment of A372V with EIPA also increased the mutation frequency from 2.5 to 4.1 mutations per 10^4^ nucleotides (*P* = 0.05) and correlated the higher titers compared to wild type virus [8]. We then examined whether amiloride treatment biased the mutation profile towards specific substitutions, as is common for base analog RNA mutagens. In untreated populations, the transition mutations AtoG and TtoC (UtoC on RNA genome) were most common (Figure 4C). Treatment of populations with ribavirin, which biases the mutation profile, resulted in the accumulation of GtoA and CtoT (CtoU on RNA) transition mutations. On the other hand, treatment with amiloride increased, but did not bias, the natural mutation profile. Taken together, our results provide the first evidence of a mutagenic activity for amiloride compounds and suggests that A372V resists this effect by increasing RdRp fidelity and lowering basal mutation frequency.

### Another high fidelity RdRp variant (poliovirus G64S) is also resistant to the mutagenic effect of amiloride

The G64S variant of poliovirus is another higher fidelity RdRp variant whose fidelity altering determinant maps to a different region of the RdRp (Figure S2). Previous studies showed that G64S was resistant to ribavirin, AZC and FU; replicated with similar kinetics to wild type poliovirus in one-step growth curves and northern blot analysis; generated virus populations with lower basal mutation frequencies and presented a higher fidelity phenotype in biochemical incorporation assays [14], [15], [16], [17], [18]. This provided the unique opportunity to determine whether amiloride exerts a mutagenic activity on a different virus with a different fidelity increasing mutation. Wild type and G64S polioviruses were treated with amiloride, and the relative mutation frequencies were determined (Figure 4D). The basal mutation frequency of wild type poliovirus was 5.8 mutations per 10^4^ nucleotides sequenced and increased to 8.2 mutations per 10^4^ nucleotides (*P* = 0.039) upon amiloride treatment. The high fidelity G64S population had a basal mutation frequency of 4.0 mutations per 10^4^ nucleotides that increased to 6.7 mutations per 10^4^ nucleotides (*P* = 0.021) in amiloride, but remained significantly lower than wild type virus (*P* = 0.05). In order to determine whether lower basal mutation frequencies correlated with resistance, amiloride treated and untreated populations were titrated (Figure 4E). A higher percentage of viruses in the G64S population survived amiloride treatment (14.3 ± 2.0%) compared to the wild type poliovirus population (1.9 ± 0.9%, *P* = 0.006). Our results therefore confirm for two different high fidelity RdRps, that increased fidelity and lower mutation frequency confer resistance to amiloride.

### Another polymerase variant of CVB3, S299T, resists amiloride mutagenic activity by a different mechanism

**Figure 5:**
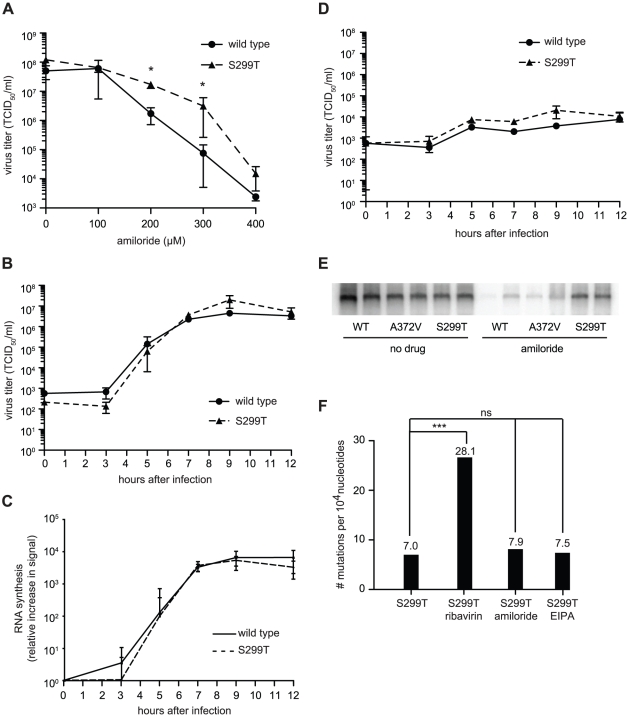
Another polymerase variant, S299T, is resistant to both the inhibitory effect of amiloride on RNA synthesis as well as its mutagenic activity. (**A**) S299T resists amiloride's antiviral activity better than wild type virus. HeLa cells were treated with different concentrations of amiloride and infected with wild type or S299T virus at an MOI of 0.01. At 48 hours, viable progeny virus was quantified by TCID_50_ assay. The mean virus titers (TCID_50_/ml) and S.E.M are shown, N = 3, * P<0.05. (**B**) One-step growth kinetics of wild type and S299T viruses. HeLa cells were infected at MOI of 10 and the progeny virus was quantified at different hours after infection by TCID_50_ assay. Mean titers (TCID_50_/ml) ± S.E.M are shown, N = 3, no significant difference found. (**C**) The relative increase in signal from time 0 to indicated time points is shown, values are means of 2 separate northern blots of samples from (B), error bars show range of values (**D**) One-step growth kinetics of wild type and S299T viruses in the presence of 400 µM amiloride. HeLa cells were infected at MOI of 10 and the progeny virus was quantified at different hours after infection by TCID_50_ assay. Mean titers (TCID_50_/ml) ± S.E.M are shown, N = 3, no significant differences found. (**E**) Northern blot analysis of RNA synthesis determined 48 hours after infection with wild type or S299T (MOI = 0.01) virus in the presence or the absence of 400 µM amiloride. Two independent treatment samples per virus are shown. (**F**) Average mutation frequencies of S299T untreated population and treated with ribavirin, amiloride or EIPA. *** P<0.0001; ns = no statistically significant difference. NB: All data from Figure 5 was obtained simultaneously with that for Figure 3 and separated for clarity of presentation, hence, wild type and A372V data is the same for both figures, except for (C).

Along with A372V, Harrison *et al*. isolated a second RdRp variant, S299T, in their screen for amiloride resistance [8]. By contrast, our screens for RNA mutagen resistance failed to isolate S299T alongside A372V. We hypothesized then that S299T is either resistant to another amiloride-mediated antiviral effect (inhibition of RNA synthesis, e.g.), or resistant to amiloride's mutagenic activity by a different mechanism. As for wild type and A372V, virus stocks were prepared from the cDNA infectious clone of S299T virus and studies were performed in parallel to wild type and A372V. Similar to A372V, S299T was more resistant than wild type at all concentrations of amiloride tested (Figure 5A). The one step-growth studies performed in the absence of amiloride showed that S299T produced virus with kinetics similar to wild type virus (Figure 5B). Likewise, northern blot analysis of these kinetic studies revealed that S299T did not produce higher levels of RNA in the absence of amiloride (Figure 5C). However, although one step growth analysis of S299T grown in the presence of amiloride also did not reveal significant differences in virus titer compared to wild type CVB3 (Figure 5D), northern blot analysis of RNA synthesized by 48 hours after infection revealed that the inhibitory effects of amiloride on S299T were not as dramatic as for wild type and A372V (Figure 5E).

Finally, to examine the potential effects of the S299T mutation on RdRp fidelity, infections were performed in the presence of ribavirin, amiloride, EIPA, or under mock-treatment and mutation frequencies were determined for each population. Unlike A372V, the mutation frequency of S299T did not suggest higher fidelity compared to wild type (Figure 5F). Rather, it presented a significantly higher mutation frequency (7.0 mutations per 10^4^ nucleotides, *P* = 0.0034), suggesting that this variant encodes a lower fidelity polymerase. Indeed, treatment with 400 µM ribavirin increased the mutation frequency to 28.1 mutations per 10^4^ nucleotides (*P*<0.0001), significantly higher than wild type (*P* = 0.0207) and A372V (*P*<0.0001) (Figure 1E) and in accordance with a lower fidelity polymerase. To confirm the lower fidelity phenotype conferred by the S299T mutation, an *in vitro* biochemical assay examining the relative levels of misincorporation with the purified S299T enzyme was performed. As shown in Figure 2, S299T RdRp incorporated more GMP over time than wild type RdRp, consistent with the S299T mutation decreasing RdRp fidelity (Table 1). Unexpectedly, when treated with either amiloride or EIPA (Figure 5F), this population did not undergo a significant increase in mutation frequency (7.9 mutations per 10^4^ nucleotides, *P* = 0.442 and 7.5, *P* = 0.859, respectively), suggesting that S299T polymerase is completely unaffected by the mechanism by which amiloride increases mutation in wild type and A372V viruses. Hence, while A372V resists the mutagenic activity of amiloride by increasing RdRp fidelity; S299T partially resists RNA replication inhibition and this mutagenic effect entirely.

### The mutagenic effect of amiloride is secondary to inhibition of RNA synthesis

**Figure 6:**
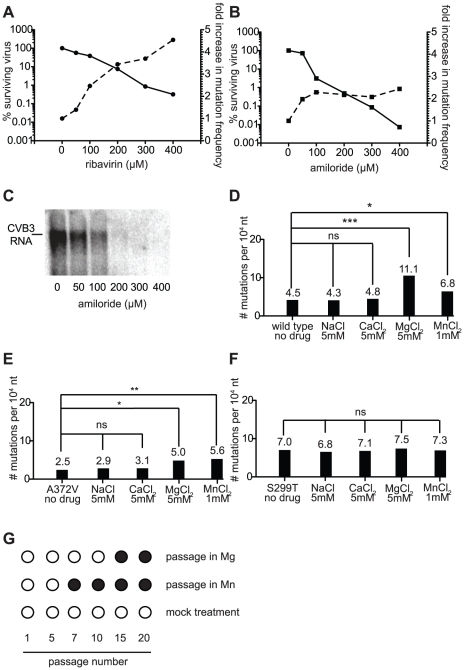
The mutagenic activity of amiloride is an indirect, secondary antiviral effect that correlates with increases in intracellular divalent cation (Mg^2+^ or Mn^2+^) concentrations. (**A**) Dose dependence of virus titer decrease and mutation frequency increase with ribavirin treatment. The reduction in wild type virus yield (solid lines, left y axis) is shown as a function of the percentage of viruses surviving treatment with different concentrations of ribavirin. The increase in mutation frequency (dashed lines, right y-axis) of the starting population at these same drug concentrations is shown. (**B**) Dose dependence of amiloride treatment, as in (A). (**C**) Inhibition of RNA synthesis by amiloride is dose dependent. Northern blot analysis of RNA synthesis of wild type virus treated with different concentrations of amiloride. Cells were infected at MOI of 0.01 and RNA was extracted 48 hours after infection. (**D**–**F**) Treatment with Mg^2+^ and Mn^2+^, but not Na^+^ or Ca^2+^, increase the mutation frequency of wild type (D) and A372V (E), but not S299T (F). Cells were infected at MOI of 0.01 with virus in media supplemented with the indicated concentrations of salts and mutation frequencies were determined in progeny populations 48 hours after infection. * P<0.05, ** P<0.01, *** P<0.0001; ns = no statistically significant difference. (**G**) Passage of wild type virus in high concentrations of Mg^2+^ and Mn^2+^ selects for high fidelity A372V. Virus was passaged 20 times in 5 mM MgCl_2_, 1 mM MnCl_2_ or regular media. At the indicated passage numbers the virus population was sequenced. The emergence of a single point mutation resulting in a A372V change is indicated by a solid circle.

Our results argue that increases in mutation frequency are a previously unknown antiviral mechanism of amiloride compounds, but the relative contribution of RNA synthesis inhibition and RNA mutagenesis is unclear. To examine the relative contribution of mutagenesis to the overall antiviral effect, we determined the mutation frequencies of wild type CVB3 virus populations treated with increasing concentrations of ribavirin or amiloride. These data were used to determine the fold increase in mutation frequency of wild type virus at increasing concentrations of drug compared to untreated control. Indeed, ribavirin treatment showed a dose-dependent increase in mutation frequency (Figure 6A, dashed line, right y-axis) that correlated with a decrease in virus titers (solid line, left y-axis). These data agree with previous work showing mutagenesis to be the principal antiviral mechanism of ribavirin in tissue culture [10]. In contrast, treatment of CVB3 with increasing concentrations of amiloride (at least above 100 µM) did not produce a corresponding increase in mutation frequency (Figure 6B, dashed line), although the decrease in virus yield was dose-dependent (solid line). Northern blot analysis of RNA synthesis at these different amiloride concentrations revealed a dose dependent effect on RNA inhibition (Figure 6C). These results suggest that replication inhibition is the principal cause of the dose-dependent drop in virus titers and that the mutagenic effects of amiloride are the result of an indirect, dose-independent effect on the polymerase.

### Increases in intracellular ion concentrations (Mg^2+^, Mn^2+^) may account for the mutagenic activity of amiloride compounds

The dose dependent decrease in viral titer (Figure 6A) attributed to inhibition of RNA synthesis is proposed to result from a direct interaction of amiloride with the RdRp [8]. Given that dose dependence of mutation frequency was not observed for amiloride, we hypothesized that this mutagenic effect was the indirect result of the effect of amiloride on the cellular environment. Indeed, previous studies have shown amiloride treatment to alter the intracellular concentrations of free Na^+^, Ca^2+^, Mg^2+^, which in turn can alter the equilibrium levels of other ions, including Mn^2+^ [1], [2], [22]. Mg^2+^ and Mn^2+^ are interesting candidates since they are cofactors essential for incorporation activity of polymerases such as viral RdRp. In our own cell culture conditions, we confirmed that in addition to the more commonly studied effects on Na^+^, Mg^2+^ levels are also significantly perturbed in cells treated with amiloride (*P* = 0.0009) (Figure S1).

To examine the potential role of altered intracellular cation concentrations in the observed mutagenic activity, wild type, A372V and S299T viruses were grown in cell media supplemented with high concentrations of either NaCl, CaCl_2_, MgCl_2_ or MnCl_2_ and the resulting mutation frequencies were determined. Growth of all three viruses in either NaCl or CaCl_2_ had no significant effect on mutation frequency (Figure 6D–F). Increasing intracellular Mg^2+^ or Mn^2+^ concentrations on the other hand, resulted in a significant increase in mutation frequency for both wild type (*P*<0.0001 for Mg^2+^, *P* = 0.046 for Mn^2+^, Figure 6D) and A372V (*P* = 0.021 for Mg^2+^, *P* = 0.0073 for Mn^2+^, Figure 6E) viruses, the frequencies of A372V being significantly lower than those of wild type (*P*<0.0001) as would be expected of the higher fidelity variant. In contrast, the mutation frequency of S299T remained unchanged (*P* = 0.695 for Mg^2+^, *P* = 0.769 for Mn^2+^, Figure 6F). Importantly, one-step growth analysis of each virus grown in the presence of this concentration of MgCl_2_ confirmed that replication itself was not affected (Figure S3). Given the similar sensitivity and resistance profiles of each variant in both these and the amiloride treated populations, our results support the notion that amiloride induced mutagenesis results from changes in intracellular concentrations of the essential divalent cation cofactors. To gather further support for the link between amiloride mutagenesis and Mg^2+^/Mn^2+^ concentrations, wild type virus was serially passaged in the presence of either compound and the RdRp regions of the passaged virus populations were sequenced at the intervals indicated (Figure 6G). Interestingly, wild type virus that was grown in the presence of MnCl_2_ had acquired (passage 5) and fixed (passage 7 onward) the same high fidelity A372V mutation that was selected by RNA mutagen (Figure 1) and amiloride [8] passage. Similarly, passage of wild type virus in MgCl_2_ selected the same high fidelity which was fixed in the population by passage 15.

## Discussion

In this report, we identify a previously undescribed mutagenic effect of amiloride treatment on CVB3 and poliovirus (Figure 4) and confirm that amiloride inhibits RNA synthesis of Coxsackie B3 virus (Figure 3E, 5E). What are the relative contributions of the two activities to the observed antiviral effect of amiloride compounds? The dose dependent inhibition of RNA synthesis (Figure 6C) that correlates with the dose dependent reduction in wild type virus titers (Figure 6B) suggests that this is the principal antiviral activity for amiloride. This may reflect a direct interaction of amiloride with the RdRp, perhaps through blocking of the nucleotide entry tunnel or catalytic site, as was suggested by Harrison *et al* [8]. More detailed *in vitro* biochemical or *in vitro* replication assays should help determine the nature of this interaction.

What is the molecular basis for amiloride induced mutagenesis? The lack of dose dependence of the observed mutagenic effect at higher concentrations of amiloride (Figure 6B) that is typically observed for RNA mutagens such as ribavirin (Figure 6A) led us to seek an indirect mechanism for amiloride induced mutagenesis. Amiloride inhibits epithelial Na^+^ channels, Na^+^/H^+^ ion antiporters and other less characterized ion exchangers (Na^+^/Ca^2+^ and Na^+^/Mg^2+^) which could in turn affect the equilibrium of other ions, such as Mn^2+^ [2], [22]. Indeed, in our own experimental conditions we observed a significant alteration of intracellular Mg^2+^ concentrations, although 800 µM amiloride treatment was necessary because of detection limits (Figure S1). Since Mg^2+^ and Mn^2+^ are essential cofactors for polymerase activity and nucleotide insertion [23], we explored a potential link between amiloride treatment, Mg^2+^/Mn^2+^ levels and CVB3 mutation frequency. Indeed, we found that increases in intracellular Mg^2+^ and Mn^2+^ correlate with increased virus mutation frequency (Figure 6D–E). Whether amiloride inhibits Mg^2+^ and/or Mn^2+^ transporters directly (which have not yet been identified in eukaryotes) or indirectly through effects on Na^+^ channels and exchangers will be difficult to determine. Studies with other channel blockers of different structure may help to pinpoint this mechanism, although redundancy between channels may mask their effect. Nevertheless, the mutagenesis and resistance profiles of the A372V and S299T amiloride-resistant variants support this link between cation concentrations and amiloride's mutagenic effect.

A372V is a higher fidelity RdRp variant that generates virus populations with lower basal mutation frequencies than wild type populations. In result, this virus population, although not impervious to the effects of mutagens, can better tolerate a moderate increase in mutation frequency that would otherwise lethally mutagenize the wild type population. Accordingly, A372V mutation frequencies increased following ribavirin treatment, amiloride treatment and treatment with high concentrations of Mg^2+^ and Mn^2+^ but in all cases, the frequencies were significantly lower than wild type populations under the same treatments. Unlike S299T, this variant is solely resistant to the mutagenic antiviral activity since its RNA synthesis is as inhibited by amiloride as wild type virus (Figure 3E). It is perhaps for this reason that passage of wild type virus in Mg^2+^ and Mn^2+^ (that mimics only the mutagenic, and not the RdRp inhibitory, activity of amiloride), resulted in the selection of the high fidelity A372V variant over S299T (Figure 6G).

The S299T variant seems to resist amiloride on both fronts: RNA synthesis is less inhibited (Figure 5E) and its mutation frequency is unchanged (Figure 5F). Whether S299T's dual mechanisms of resistance to both of amiloride's antiviral activities are tightly coupled, or coincidental, remains to be determined. Nevertheless, a similar lack of mutagenic effect by Mg^2+^ and Mn^2+^ treatment (Figure 6F) provides further support for the role of divalent cations in amiloride-induced mutagenesis. However, this seems to come at the cost of decreasing overall RdRp fidelity, as evidenced by its hypersensitivity to the nucleoside mutagen ribavirin and *in vitro* biochemical data (Figure 2 and 5F). Interestingly, low fidelity variants of poliovirus and FMDV also map to the same β9–α11 loop, a domain interacting with the active site where coordination of one of two divalent cation cofactors and the incoming nucleotide occurs [24], [25]. Alterations to this domain may alter RdRp dependence on the relative availability of the Mg^2+^ cofactor, thereby resulting in a lower fidelity, Mg^2+^ concentration-insensitive RdRp or may change its preference for Mg^2+^ to Mn^2+^. In poliovirus, a position 297 variant was shown to be a lower fidelity RdRp *in vitro* and a position 296 variant of FMDV also presented higher mutation frequencies [24], [26]. In earlier studies, position 297 poliovirus variants were found to be dependent on Mn^2+^ for growth[27]. Biochemical assays have shown that poliovirus RdRp activity in the presence of Mn^2+^ results in higher mutation frequencies[28], [29] and if S299T is Mn^2+^ dependent, it may explain the higher basal mutation frequency relative to wild type virus.

The use of RNA mutagens to extinguish viral populations by hypermutation is a promising antiviral approach [11], [12], [13], [19], [30], [31], [32]. Lower fidelity variants (such as S299T) could more readily identify weakly mutagenic base analogs that could later be improved, while higher fidelity variants (such as A372V) would help test the efficacy of the strongest of mutagens, of nucleoside or non-nucleoside structure [33], [34], [35]. Since the description of lethal mutagenesis using base analogs, there is speculation as to whether other compounds can alter mutation frequency through direct or indirect action on the RdRp. It is important to note that the mutagenic activity of amiloride observed in our study was not as significant as that observed for nucleoside RNA mutagens. This raises a two-sided question: is the activity of amiloride strong enough to lethally mutagenize the virus population over a prolonged exposure or would the moderate increase in mutation frequency help the virus more rapidly evolve or escape immune responses? Under these experimental conditions, the mutagenic effect was strong enough to force the virus to develop two mechanisms of resistance, suggesting that this mutagenic activity alone was sufficiently detrimental to the virus. Whether the antiviral mutagenic effect we observed here occurs at physiological intracellular amiloride concentrations in humans is not yet known and how this may affect the fidelity of cellular RNA polymerases remains to be studied. Nevertheless, our work uncovers a new target for drug discovery - compounds that induce reversible alterations of the intracellular concentrations of essential cation cofactors for viral RdRp. Our results should encourage screening of compound libraries for new molecules that, through direct interaction with RdRps or indirect effects on the intracellular environment, may alter the mutation frequency of RNA viruses by modulating polymerase fidelity.

Finally, our study identifies two new CVB3 RdRp fidelity variants. To date, only position 64 variants of poliovirus were shown to exhibit increased RdRp fidelity [15], [18]. Despite having similar RdRp structures [36], [37], [38], A372V maps to a distant region of the polymerase (Figure S2). Our previous work and current findings (higher fidelity A372V and lower fidelity S299T), along with the description of lower fidelity RdRp of poliovirus and Foot and Mouth Disease virus [24], [26], suggest that the intrinsic fidelity of RdRps is defined by multiple residues. The full extent of this fidelity network and whether it translates across RdRps from different virus families is yet to be determined. In addition to their utility in studies such as our current report, RdRp variants are valuable tools to study viral evolution and adaptation of RNA virus populations *in vivo* [14], [16], [17]. It will be interesting to determine how a virus population of increased or decreased genetic diversity will behave in the context of Coxsackie virus infection.

## Methods

### Cells, plasmids, drugs

HeLa (Young) and Vero cells were maintained in DMEM medium with 10% newborn calf serum. Plasmid bearing the cDNA of Coxsackie virus B3 (Nancy) strain was a kind gift of F. van Kuppeveld (Radboud University, Nijmegen, Netherlands). Plasmids containing poliovirus cDNAs were previously described [17]. The following compounds were obtained from Sigma Aldrich:

Ribavirin IUPAC 1-[(2R,3R,4S,5R)-3,4-dihydroxy-5-(hydroxymethyl)oxolan-2-yl]-1H-1,2,4-triazole-3-carboxamide);

5-fluorouracil IUPAC 5-fluoro-1H-pyrimidine-2,4-dione;

5-Azacitidine IUPAC 4-amino-1-β-D-ribofuranosyl-1,3,5-triazin-2(1H)-one;

Amiloride IUPAC 3,5-diamino-6-chloro-N-(diaminomethylene)pyrazine-2-carboxamide;

EIPA IUPAC 3-amino-6-chloro-N-(diaminomethylidene)-5-[ethyl(propan-2-yl) amino]pyrazine-2-carboxamide;

MIA IUPAC 3-amino-5-[tert-butyl(methyl)amino]-6-chloro-N-(diaminomethylidene)pyrazine-2-carboxamide;

benzamil IUPAC 3,5-diamino-N-(N'-benzylcarbamimidoyl)-6-chloropyrazine-2-carboxamide.

### Generation of virus stocks by *in vitro* transcription and electroporation

All studies were performed on virus stocks (wild type, A372V or S299T) generated from cDNA infectious clones. The A372V and S299T variants were constructed using the Quikchange XL site directed mutagenesis kit (Stratagene) and the CVB3-Nancy infectious cDNA. Three infectious cDNA clones of each variant (A372V and S299T) were obtained, sequenced and used to generate three independent virus stocks. Three virus stocks of wild type virus were also prepared in this manner. Each of three stocks served as one of three triplicate samples in replication studies, and RNA mutagen and amiloride compound sensitivity assays. For mutation frequency data, the virus population generated by clone 1 for each variant was used throughout the study. Basal mutation frequencies were determined on 2 independently generated samples of each variant and no statistically significant differences in mutation frequencies were found. CVB3 cDNA plasmids were linearized with *Sal* I and poliovirus cDNA plasmids, with *Eco* RI. Linearized plasmids were purified with the Qiagen PCR purification kit. 2.5 µg of linearized plasmid was *in vitro* transcribed using T7 RNA polymerase (Fermentas). 8 µg of transcript was electroporated into 4×10^6^ Vero cells that were washed twice in PBS (w/o Ca^2+^ and Mg^2+^) and resuspended in PBS (w/o Ca and Mg) at 10^7^ cells/ml. Electroporation conditions were as follows: 0.4 mm cuvette, 25 µF, 700 V, maximum resistance, exponential decay in a Biorad GenePulser XCell electroporator. Cells were recovered in DMEM. 500 µl of p0 virus stock was used to infect 3×10^6^ Vero cells in T25 flasks, to produce p1 virus. 250 µl of p1 virus was used to infect 1×10^7^ Vero cells in DMEM-10% NCS in T75 flasks, to produce p2 virus. For each passage, virus was harvested at total cytopathic effect (CPE) by one freeze-thaw cycle and clarified by spinning at 10 K rpm for 10 minutes.

### Determination of viral titers: By TCID_50_

Ten-fold serial dilutions of virus were prepared in 96-well round-bottom plates in PBS. Dilutions were performed in octuplate and 100 µl of dilution were transferred to 10^4^ Vero cells plated in 100 µl of DMEM. After 5 days living cell monolayers were colored by crystal violet. TCID_50_ values were determined by the Reed and Muensch method. **By plaque assay**. HeLa cells were seeded into 6-well plates and virus preparations were serially diluted (10-fold) in PBS. Cells were washed twice with PBS and infected with 250 µl of dilution for 30 minutes at 37°C, after which a semisolid overlay comprised of DMEM medium and 1.2% w/v Avicell (FMC Biopolymer) was added. 2 days after infection, cells were washed and stained with crystal violet 0.2%, and plaques were enumerated.

### Isolation of RNA mutagen and divalent cation resistant viruses

HeLa cell monolayers were pretreated with 50 µM ribavirin, 50 µM 5-Azacytidine, 5 mM MgCl_2_ or 1 mM MnCl_2_ for 2 hours, then infected with 10^6^ TCID_50_ of CVB3. Blind serial passages were then performed on fresh mutagen-treated HeLa cell monolayers (250 µl of virus-containing supernatant). At the indicated passage intervals, viral RNA was extracted from purified virions with Trizol reagent (Invitrogen) and RT-PCR was performed (Titan One-Step, Roche). PCR products were sequenced to identify consensus sequence changes within the RdRp region, between nucleotides 4701 and 7903.

### RNA mutagen, amiloride compound and cation assays

HeLa cell monolayers in 6-well plates were pretreated for 2 hours (ribavirin, AZC, FU, NaCl, CaCl, MgCl_2_, or MnCl_2_) or 10 hours (amiloride compounds) with different concentrations of compound as indicated. We chose and verified concentrations of compounds that were not toxic to cells over a 72 hours period. For amiloride compounds, we chose and confirmed concentrations corresponding to virus inhibitory concentration (IC_50_) values that were not toxic to cells, as determined by Harrison et al.[8]. Cells were then infected at an MOI = 0.01 with passage 2 virus. 48 hours post-infection, virus was harvested by one freeze-thaw cycle and virus titers (TCID_50_ or plaque assay) were determined.

### Replication kinetics

For replication studies, HeLa cells were either pretreated with 400 µM amiloride or mock treated and infected at an MOI of 10. For one-step growth kinetics, transfected cells were frozen at different time points after infection and later titered by TCID_50_ assay. For Northern blot analysis, total RNA from infected cells was extracted by Trizol reagent (Invitrogen) and purified. 5 µg of total RNA were used per sample (measured by Nanodrop). Gels were transferred onto a nitrocellulose membrane (Whatman Turboblotter SuperCharge Nylon membrane kit), hybridized overnight with a dCTP-α^32^P labeled DNA probe corresponding to 200 bp of the RdRp, visualized on a Storm Phosphorimager and analyzed by ImageQuant.

### Determination of mutation frequency by sequencing

At total CPE, viral RNA in supernatants was extracted and RT-PCR amplified using the primers sets 878Forward and 2141Rev for CVB3 virus and 1337For and 2651Rev for poliovirus. The resulting PCR products were purified on column (Nucleospin, Macherey-Nagel) and TopoTA cloned (Invitrogen). Blue/white screening was used on single colony transformants, positive clones were sequenced (GATC Biotech). Sequence data was analyzed using the Lasergene software package (DNAStar Inc). For statistical purposes, we retained sequence data only over the region for which every clone was represented (859 nucleotides for CVB3 and 884 nucleotides for PV). The number of mutations per 10^4^ nucleotides sequenced was determined using the total mutations identified per population over the total number of nucleotides sequenced for that population multiplied by 10^4^. For each population, between 68 and 178 clones were sequenced representing between 58,000 to 153,000 nucleotides per sample (see Table S1). If the same mutation recurred in the same population, this was only counted once; however, this only occurred in two instances of one single repetition each and was not sufficient to change the mutation frequency values and statistics.

### Cytosolic Mg^2+^ measurements in HeLa cells following amiloride treatment

A confluent layer of HeLa cells was treated with 800 µM amiloride for 10 hours and incubated with PBS containing 100 µM Ethylene glycol-bis (β-aminoethyl ether)-N,N,N',N'-tetraacetic acid (EGTA) for 15 min, to chelate Ca^2+^, before lysis in H2O-Tween 0.1%. The ratiometric magnesium indicator mag-fura-2 (Invitrogen) was added to a final concentration of 2 µM to 1 ml of sample and fluorescence was measured at 25°C using a Quanta-Master QM4CW spectrofluorometer (PTI) using a 1 cm path length quartz cuvette thermostated at 25°C. Excitation scans were performed from 250 to 490 nm with 1 nm steps, using 1 nm bandwidth; emission was monitored at 530 nm with 5 nm bandwidth. Continuous recordings of fluorescent intensities at 330 nm and 370 nm were transformed into 330/370 wavelength ratios.

### Statistical analyses

For one step growth curves, mutagen treatment assays and Mg^2+^concentration assays, the two-tailed paired student's t tests were used to determine significance with 95% confidence intervals. For mutation frequencies, χ^2^ tests and two-tailed Mann Whitney U tests were performed. χ^2^ tests compared the total number of mutations in a population to total nucleotides sequenced. Mann Whitney tests compared the ranked scores of number of mutations found in individual clones grouped by population. In all cases, Mann Whitney tests gave the more conservative *P* values and are the indicated here. Statistics were performed using Prism software (GraphPad Inc).

### In vitro biochemical assay for measuring RdRp fidelity

All RNA oligonucleotides were from Dharmacon Research, Inc.; [γ-^32^P]-ATP (7000 Ci/mmol) was from MP Biomedical; T4 polynucleotide kinase was from USB; ATP and GTP were from GE Healthcare; all other reagents were of the highest grade available from Sigma, Fisher or VWR. RNA oligonucleotides were purified by denaturing PAGE and end-labeled by using [γ-^32^P]ATP and T4 polynucleotide kinase as described previously[21]. Concentrations were determined by measuring the absorbance at 260 nm using a Nanodrop spectrophotometer and using the appropriate calculated extinction coefficient.

### Construction, expression and purification of wild type, A372V and S299T CVB3 RdRp

Expression constructs for wild type, A372V and S299T CVB3 RdRp were created by using standard recombinant DNA protocols. DNA sequences were amplified using the appropriate CVB3 cDNA as template. Forward and reverse primers employed for amplification were selected based on the presence of unique restriction sites suitable for cloning of the 3D gene (RdRp) into the pSUMO expression plasmid[39] *E. coli* Rosetta cells were tansformed with the appropriate plasmid and these cells were used to produce an inoculum for large-scale growth. CVB3 3D gene expression was induced during exponential growth by addition of isopropyl-β-D-thiogalactopyranoside or by using auto-induction[40]. Induced cells were lysed in appropriate buffers, and the enzymes were purified to apparent homogeneity by using standard column chromatography resins and protocols[39].

### In vitro nucleotide incorporation assays

Reactions were performed at 30°C. ^32^P-labeled primer extension assays: 2 µM CVB3 RdRp was mixed with 0.5 µM [^32^P]-primer-template substrate in 50 mM HEPES pH 7.5, 5 mM MgCl_2_, 60 µM ZnCl_2_ and 10 mM 2-mercaptoethanol. Reactions were initiated by the addition of ATP or GTP (1 or 5 mM) in 50 mM HEPES pH 7.5, 5 mM MgCl_2_, 60 µM ZnCl_2_, 10 mM 2-mercaptoethanol and 200 mM NaCl. Reactions were quenched at various times by addition of quench buffer (50 mM EDTA, 70% formamide, 0.025% bromphenol blue, and 0.025% xylene cyanol). CVB3 RdRp was diluted immediately prior to use in 50 mM HEPES pH 7.5, 20% glycerol and 10 mM 2-mercaptoethanol. The volume of enzyme added to any reaction was always less than or equal to one-tenth the total volume. Products were resolved by denaturing PAGE. Stopped-flow fluorescence assay: Pre-steady state **s**topped-flow fluorescence experiments were performed using a Model SF-2001 stopped-flow apparatus (Kintek Corp., Austin, TX) equipped with a waterbath. All reactions were performed at 30°C. 2 µM CVB3 3Dpol was mixed with 0.5 µM primer-template substrate containing 2-aminopurine ribonucleoside on the 5′ side of the templating base[41] in 50 mM HEPES pH 7.5, 5 mM MgCl_2_, 60 µM ZnCl_2_ and 10 mM 2-mercaptoethanol. Reactions were initiated by the addition of 1 mM ATP in 50 mM HEPES pH 7.5, 5 mM MgCl_2_, 60 µM ZnCl_2_, 10 mM 2-mercaptoethanol and 200 mM NaCl. After mixing, reactant concentrations were reduced by 50%. Fluorescence emission was monitored by using a 370 nm cut-on filter (model E370LP, Chroma technology corp., Rockingham, VT.). The excitation wavelength used was 313 nm. For each experiment, at least four fluorescence traces were averaged. The relative fluorescence was plotted as a function of time and fit to a single exponential equation, 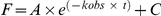, where F is the relative fluorescence intensity, kobs is the observed rate constant for nucleotide incorporation, t is the time and C is an offset.

### Product analysis, denaturing PAGE

Quenched reaction mixtures were heated to 70°C for 2–5 min prior to loading 5 µl on a 20% denaturing polyacrylamide gel containing 1X TBE and 7 M urea. Electrophoresis was performed in 1X TBE at 85 watts. Gels were visualized by using a PhosphorImager and quantified by using the ImageQuant software (Molecular Dynamics). Data were fit by nonlinear regression using the program, KaleidaGraph (Synergy Software, Reading, PA).

## Supporting Information

Figure S1 Amiloride treatment of cells increases free intracellular Mg^2+^ concentrations. HeLa cells were treated for 10 hours with 800 µM or without amiloride, washed, and lysed. Increases in intracellular Mg^2+^ concentrations were determined by the change in the ratio of emission signals at 330/370 nm using mag-fura-2 indicator. The mean values and S.E.M. are shown. * P = <0.05, N = 6.
(0.39 MB EPS)

Figure S2 Localization of fidelity altering mutations in picornavirus RdRp. The crystal structure of the CVB3 RdRp [36], [37] is shown, in color format progressing from N terminus (blue) to C terminus (red). The position 64 shown to increase the fidelity of poliovirus RdRp, position 299 that decreases fidelity of CVB3 RdRp and position that increases fidelity of CVB3 RdRp are indicated and illustrated in ball structure. The location of the catalytic site of the picornavirus RdRp is also indicated and represented in line structure.
(3.00 MB EPS)

Figure S3 Replication of wild type, A372V and S299T is not affected by increasing the concentration of MgCl_2_ in the media. One-step growth kinetics of wild type A372V and S299T viruses were performed in the presence of 5 mM MgCl_2_. HeLa cells were infected at MOI of 10 and the progeny virus was quantified at different hours after infection by TCID_50_ assay. Mean titers (TCID_50_/ml) ± S.E.M are shown, N = 3, no significant difference found.
(0.50 MB EPS)

Table S1 Mutation distribution data for experiments presented.
(0.08 MB PDF)
