# Supplementary material for: Fidelity Variants of RNA Dependent RNA Polymerases Uncover an Indirect, Mutagenic Activity of Amiloride Compounds
Source: PLoS Pathog. 2010 Oct 28;6(10):e1001163. doi: 10.1371/journal.ppat.1001163 (PMC2965762; doi:10.1371/journal.ppat.1001163)
Supplement: Table S1 — Mutation distribution data for experiments presented. (0.08 MB PDF) [file ppat.1001163.s004.pdf]

Table S1: Mutation distribution data for experiments presented.

# clones with n mutations	WT	A372V	S299T	WT ribavirin	A372V ribavirin	S299T ribavirin
7 mutations	0	0	0	1	0	3
6 mutations	0	0	0	1	1	2
5 mutations	0	0	0	2	0	7
4 mutations	0	0	0	5	3	4
3 mutations	1	0	5	9	6	10
2 mutations	6	2	11	15	15	18
1 mutations	40	14	70	25	29	14
0 mutations	95	68	92	13	20	10
**Total mutations**	55	18	107	125	95	164
**Total # clones**	142	84	178	71	74	68
**Total nt sequenced**	121,978	72,156	152,902	60,989	63,566	58,412
**Mutations/10^4 nt**	**4.51**	**2.49**	**7.00**	**20.50**	**14.94**	**28.08**

# clones with n mutations	WT amiloride	A372V amiloride	S299T amiloride	WT EIPA	A372V EIPA	S299T EIPA
7 mutations	0	0	0	0	0	0
6 mutations	0	0	0	0	0	0
5 mutations	1	0	0	0	0	0
4 mutations	0	1	1	0	0	0
3 mutations	10	2	2	4	1	4
2 mutations	15	9	13	10	3	8
1 mutations	38	39	29	27	16	24
0 mutations	64	75	48	40	51	45
**Total mutations**	103	67	65	59	25	52
**Total # clones**	128	126	93	81	71	81
**Total nt sequenced**	109,952	108,234	79,887	69,579	60,989	69,579
**Mutations/10^4 nt**	**9.37**	**6.19**	**7.89**	**8.48**	**2.49**	**7.47**

# clones with n mutations	WT MIA	WT benzamil	polio WT	polio G64S	polio WT amiloride	polio G64S amiloride
7 mutations	0	0	0	0	0	0
6 mutations	1	0	0	0	0	0
5 mutations	0	0	0	0	0	0
4 mutations	1	0	0	0	0	0
3 mutations	3	1	2	0	6	4
2 mutations	12	7	10	5	12	10
1 mutations	14	26	36	35	34	34
0 mutations	36	45	74	88	46	64
**Total mutations**	57	43	62	45	72	66
**Total # clones**	67	79	122	128	96	112
**Total nt sequenced**	57,553	67,861	107,848	113,152	84,864	99,008
**Mutations/10^4 nt**	**9.90**	**6.34**	**5.75**	**3.97**	**8.48**	**7.47**

# clones with n mutations	WT NaCl	A372V NaCl	S299T NaCl	WT $CaCl_2$	A372V $CaCl_2$	S299T $CaCl_2$
7 mutations	0	0	0	0	0	0
6 mutations	0	0	0	0	0	0

5 mutations	0	0	0	0	0	0
4 mutations	0	0	0	0	0	0
3 mutations	0	0	3	0	0	1
2 mutations	4	1	10	4	0	13
1 mutations	36	27	41	40	30	37
0 mutations	54	64	40	42	64	41
**Total mutations**	44	29	70	48	30	66
**Total # clones**	94	92	94	90	94	92
**Total nt sequenced**	103,400	101,200	103,400	99,000	103,400	92,000
**Mutations/10^4 nt**	**4.26**	**2.87**	**6.77**	**4.85**	**2.90**	**7.17**

**# clones with n mutations**	**WT $MgCl_2$**	**A372V $MgCl_2$**	**S299T $MgCl_2$**	**WT $MnCl_2$**	**A372V $MnCl_2$**	**S299T $MnCl_2$**
7 mutations	1	0	0	0	0	0
6 mutations	0	0	0	0	0	0
5 mutations	2	0	3	0	0	0
4 mutations	2	0	0	1	0	1
3 mutations	4	1	1	0	1	0
2 mutations	15	8	12	11	5	15
1 mutations	35	20	26	21	37	25
0 mutations	37	53	52	22	47	40
**Total mutations**	102	39	68	47	48	59
**Total # clones**	96	82	94	69	90	81
**Total nt sequenced**	92,160	78,720	90,240	69,000	90,000	81,000
**Mutations/10^4 nt**	**11.07**	**4.95**	**7.53**	**6.81**	**5.56**	**7.28**
